# Correction of patient positioning errors based on in-line cone beam CTs: clinical implementation and first experiences

**DOI:** 10.1186/1748-717X-1-16

**Published:** 2006-05-24

**Authors:** Christoph Thilmann, Simeon Nill, Thomas Tücking, Angelika Höss, Bernd Hesse, Lars Dietrich, Rolf Bendl, Bernhard Rhein, Peter Häring, Christian Thieke, Uwe Oelfke, Juergen Debus, Peter Huber

**Affiliations:** 1 Dept. of Radiooncology, German Cancer Research Center, Heidelberg, Germany; 2 Dept. of Medical Physics, German Cancer Research Center, Heidelberg, Germany; 3 Clinical Radiology, University of Heidelberg, Heidelberg, Germany

## Abstract

**Background:**

The purpose of the study was the clinical implementation of a kV cone beam CT (CBCT) for setup correction in radiotherapy.

**Patients and methods:**

For evaluation of the setup correction workflow, six tumor patients (lung cancer, sacral chordoma, head-and-neck and paraspinal tumor, and two prostate cancer patients) were selected. All patients were treated with fractionated stereotactic radiotherapy, five of them with intensity modulated radiotherapy (IMRT). For patient fixation, a scotch cast body frame or a vacuum pillow, each in combination with a scotch cast head mask, were used. The imaging equipment, consisting of an x-ray tube and a flat panel imager (FPI), was attached to a Siemens linear accelerator according to the in-line approach, i.e. with the imaging beam mounted opposite to the treatment beam sharing the same isocenter. For dose delivery, the treatment beam has to traverse the FPI which is mounted in the accessory tray below the multi-leaf collimator. For each patient, a predefined number of imaging projections over a range of at least 200 degrees were acquired. The fast reconstruction of the 3D-CBCT dataset was done with an implementation of the Feldkamp-David-Kress (FDK) algorithm. For the registration of the treatment planning CT with the acquired CBCT, an automatic mutual information matcher and manual matching was used.

**Results and discussion:**

Bony landmarks were easily detected and the table shifts for correction of setup deviations could be automatically calculated in all cases. The image quality was sufficient for a visual comparison of the desired target point with the isocenter visible on the CBCT. Soft tissue contrast was problematic for the prostate of an obese patient, but good in the lung tumor case. The detected maximum setup deviation was 3 mm for patients fixated with the body frame, and 6 mm for patients positioned in the vacuum pillow. Using an action level of 2 mm translational error, a target point correction was carried out in 4 cases. The additional workload of the described workflow compared to a normal treatment fraction led to an extra time of about 10–12 minutes, which can be further reduced by streamlining the different steps.

**Conclusion:**

The cone beam CT attached to a LINAC allows the acquisition of a CT scan of the patient in treatment position directly before treatment. Its image quality is sufficient for determining target point correction vectors. With the presented workflow, a target point correction within a clinically reasonable time frame is possible. This increases the treatment precision, and potentially the complex patient fixation techniques will become dispensable.

## Introduction

A CT scan acquired for treatment planning usually represents only a single snapshot of the anatomical structures in time and is gathered several days before treatment. The shape and location of internal soft tissue structures at the time of treatment may deviate considerably from the initial scan. This problem cannot be solved by further improvements of external patient positioning like more rigid fixation devices. Especially in high precision radiotherapy, the daily position of the target needs to be confirmed before irradiation by a reliable imaging modality.

Different approaches are available for three-dimensional image acquisition inside the radiation treatment room. Megavoltage CT and kilovoltage CT (helical and cone beam) has been tested so far [1-3]. Kilovoltage CT has become the standard modality for soft tissue identification and target definition in conformal radiation therapy. A well established approach for in-room image acquisition is the use of a conventional CT scanner sharing the same couch with the linear accelerator [4], e.g. the Siemens PRIMATOM system (Siemens OCS, Concord, USA) combining the linear accelerator Siemens Primus and the CT scanner Siemens Emotion. The advantage of that system obviously is that all components are separately established for clinical application. The achievable high image quality and the accuracy of the system allow a reasonable handling of interfractional setup errors and organ motion. However, besides the disadvantage of having two large technical systems in a radiotherapy bunker, such systems cannot detect intrafraction motion. Also the necessary repositioning of the patient between the CT scan and the irradiation adds time to the overall procedure.

Using an in-line imaging setup attached to the gantry of the linear accelerator allows to overcome these disadvantages. Such an equipment consisting of an x-ray tube and a flat panel imager (FPI) attached to a linear accelerator (LINAC) (Siemens OCS) is available in our institution. The purpose of the present study was the clinical implementation of the kV cone beam CT (CBCT) and its application for patient setup correction in radiotherapy (RT). The paper focuses on the development of a reliable workflow from image acquisition to correction of interfraction setup deviations. We will also discuss further improvements and the potential clinical impact.

## Patients and methods

### Patients, image acquisition in treatment position

For evaluation of the setup correction workflow, six tumor patients were selected. Two of them suffered from localized prostate cancer, the remaining from lung cancer, sacral chordoma, head and neck and paraspinal tumors. The patient characteristics are summarized in table 1.

**Table 1:** Patient characteristics

**Patient number**	**diagnosis**	**target volume**	**treatment technique**	**fixation**
#1	lung cancer cT2cN0 right lower lobe	primary tumor (boost)	fractionated stereotactic radiotherapy	vacuum pillow
#2	oropharyngeal cancer pT2cN0	primary and locoregional lymph nodes	fractionated IMRT	vacuum pillow and head mask
#3	prostate cancer T3c Gleason score 6 PSA 5.6	prostate and seminal vesicles	fractionated IMRT	stereotactic body cast and head mask
#4	prostate cancer T2c Gleason score 7 PSA 12.0	prostate and seminal vesicles	fractionated IMRT	stereotactic body cast and head mask
#5	unresectable chordoma	lumbosacral spine	fractionated IMRT	stereotactic body cast and head mask
#6	recurrence of soft tissue sarcoma	lumbal spine and right m. psoas	fractionated IMRT	stereotactic body cast and head mask

All patients were treated with fractionated stereotactic RT. All except the lung cancer patient were treated with IMRT. Every patient was treated in an individually customized fixation device. Patients with prostate cancer and paraspinal tumors were immobilized by a wrap-around body cast and a head mask. For treatment of the thoracic and head-and-neck-region, a vacuum pillow was used. Both extracranial fixation devices were complemented by a head mask to eliminate head rotations which might translate into movements of the spine. Both systems were embedded in a stereotactic frame enabling stereotactic image correlation [5].

Dose plans for both IMRT and conventional treatment planning in 3D conformal technique (CRT) were calculated using the treatment planning system Voxelplan [6]. Inverse treatment planning for IMRT was carried out with KonRad™ [7]. The dose delivery of the IMRT fields was carried out in the step-and-shoot technique. Beam shaping was calculated for a 6 MV LINAC fitted with a multi-leaf collimator with 10 mm leaf width.

In daily clinical routine we normally use the available in-room CT scanner of the PRIMATOM to detect and, if necessary, correct for interfractional setup errors. For the study presented in this paper, we installed the in-line imaging equipment onto the linear accelerator of the PRIMATOM system.

### Imaging system and acquisition

The integrated imaging system presented in this paper consists of a kV x-ray tube (Siemens "Optitop") and a flat panel radiation image detector (FPI) from PerkinElmer (XRD 1640) attached to the Primus LINAC following the in-line approach. For this approach the diagnostic kV x-ray tube is mounted at an angle of 180 degree with respect to the therapeutic treatment beam (fig. 1). Both technical components (x-ray tube and FPI) are attached to the linear accelerator by in-house developed devices. The x-ray tube position was chosen to have the same source-to-isocenter distance (SID) as the treatment beam, i.e., SID = 100 cm. The distance from the kV-source to the front plane of the detector is approximately 140 cm. Therefore the central axis of the kV-imaging beam is always aligned with the central axis of the MV therapy beam. Another important feature of the in-line geometry is that the FPI can take images using the kV- and the MV-beam at the same time. This enables the online validation of the delivered fluence to the patient and the possibility to calculate the dose actually delivered to the patient [8].

**Figure 1:**
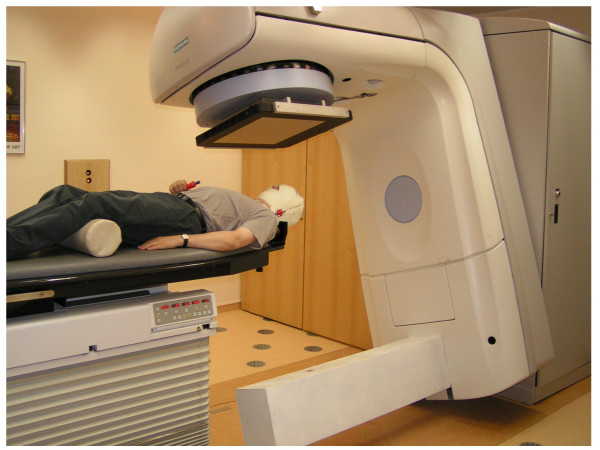
Linear accelerator equipped with an x-Ray tube mounted at the opposite side of the MV-beam source. The flat panel detector is attached right below the multi-leaf collimator. Single kV-images or cone beam CT sequences of patient in treatment position can be acquired for image guide radiotherapy.

The impact of the panel on the dose distribution was evaluated carefully prior to the treatment of the patients. The monitor units must be scaled by a factor of 1.18 to obtain the same dose inside the patient as without the FPI in the accessory holder. No impacts on the depth dose distribution, lateral profiles or dose to the patient's surface were found.

The selected x-ray tube features a 40 kW-0.6 mm and an 80 kW-1 mm focal spot, 150 kVp nominal voltage and has a 12 degree anode target angle. The imaging detector has an active area of about 40.96 × 40.96 cm^2^, a spatial resolution of 0.4 mm in each direction for a 1024 × 1024 bixel-matrix with 16 bit gray values. The detector uses a Gd_2_O_2_S:Tb scintilator and the fastest readout time is about 66 ms.

The imaging control system is located within the control room of the linac next to the treatment console. Through this control system, the user can select different imaging modes like the acquisition of single x-ray pulses, sequences of different x-ray pulses or fluoroscopy imaging acquired on external trigger signals. For each x-ray pulse the user can define values for the tube current (mA), the pulse length (ms) and the high voltage value (kVp). These parameters are then transferred to the x-ray hardware control system. Typical acquisition parameters for a projection image were 120 kVp, 20 ms and 50 mA. For 200 projections this leads to dose of 14 mGy at the isocenter of a cylindrical water phantom with a diameter of 18 cm.

To acquire a cone beam CT, the gantry of the linear accelerator rotates around the patient in treatment position at a fixed speed. An inclinometer attached to the linac's gantry generates a trigger signal for gantry angles with a fixed angle increment. This signal finally generates the x-ray pulse for one CT-image projection which is directly transferred from the detector to the reconstruction computer for further image processing.

The raw images obtained from the detector are then corrected for pixel based dark image offset and detector gain structure as well as for corrupt pixels and x-ray field inhomogeneities. These corrections are performed with the help of previously stored offset and gain correction images. The offset images were acquired directly prior to the patient images, while only one gain image was acquired in the morning [9].

A geometrical calibration is necessary due to mechanical flexibility of the x-ray tube holder and the FPI during gantry rotation. This is done using a cylindrical calibration phantom with regularly placed bullets on a helical trajectory at its periphery. The detailed calibration procedure is described in the thesis of M. Ebert [10].

The processed images and the calibration data are transferred to an in-house developed reconstruction tool using the standard Feldkamp-David-Kress (FDK) algorithm for cone beam CT reconstruction [11]. The output is a 3D CT-dataset with user specified voxel resolution. For all cases, 256 × 256 × 256 voxels with a resolution of 1.0 mm were reconstructed except for the prostate cases where a voxel resolution of 1.56 mm was chosen due to the larger field of view. The reconstruction time for a cone beam data set varies depending on the selected resolution and the number of used projections. Typically it is between 1 and 3 minutes on a 3 GHz personal computer.

To reconstruct a complete 3D data set of a cone beam CT, projections over a range of at least 200 degrees (180 degree + two times the fan beam angle)(Ref auf Ebert) must be acquired. This procedure is called "short scan". We used a spacing of one degree and therefore acquired 200 projections per patient.

### Detection and correction of setup errors

The workflow schematically shown in figure 2 was used to detect and correct for any misalignment of the target volume in the described clinical cases (fig. 2). The first steps are the patient positioning, the image acquisition and the reconstruction of the 3D data set as described in the previous section. The next step is the rigid registration of the acquired cone beam CT with the diagnostic planning CT. This is achieved by either manually selecting bony landmarks or by using an automatic matching algorithm that maximizes mutual information. The result of the mutual information matching is determined by all grey values and not restricted to the bones. The successful registration of the two datasets is approved by a visual comparison of clearly identifiable landmarks (e.g. bony structures) within both image sets. With the information now available, the dislocation of the tumor target volume can be calculated. Thereby the target volume is treated as a rigid body, i.e., its new position in space is determined by a rigid transformation with 6 degrees of freedom (a 3-dimensional spatial translation and the 3 Euler rotations around the axes through the isocenter). Deformations of the target were not accounted for. Only the target translations could be used for the target positioning process, in which the translation vector of the target is converted to a respective shift of the treatment table. The rotational error was documented, but could not be corrected for. The offset values between the original and the new table position were automatically transferred to the treatment table and the shift was then automatically executed under the supervision of the technician and a physician.

**Figure 2:**
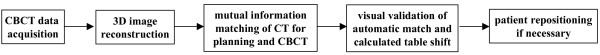
Schematic description of the workflow applied for automatic patient positioning.

For the translations an action level of 2 mm for each axis of the translation vector was defined. The threshold for the rotation angles and the transversal shift vector were derived from the applied safety margin to the CTV during the planning process. Only if the offset components were larger than the action level, the patient was shifted to the new treatment position.

The residual error after the correction of the transversal components is mainly given by the positioning precision of the table which is +-0.5 mm. Additional intrafractional variations also contribute to the remaining error, however, these were not analyzed in the present study.

## Results and discussion

### Matching and image quality

Figure 3 shows exemplary CT slices of the planning CT and the CBCT for the lung, head-and-neck, prostate and paraspinal cases. In all cases, the automatic matching algorithm could register the CBCT to the planning CT. The registration was verified by visual assessment of clearly identifiable bony landmarks which showed an exact match.

**Figure 3:**
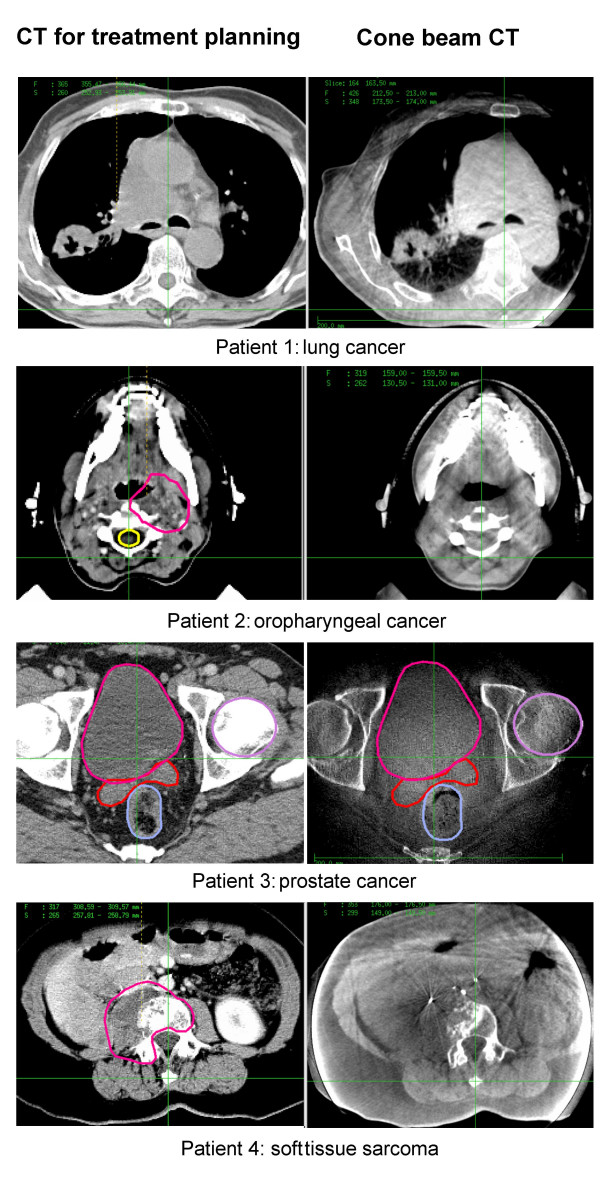
Clinical examples of cone beam (right side) compared to diagnostic treatment planning CT (left side).

At the current stage of development, the overall image quality and especially the soft tissue contrast of the CBCT scans do not reach the standard of dedicated diagnostic CT scans. The reduced contrast is partly due to scattered photons. For larger patients the distance between the object to image and the detector is reduced and therefore the percentage of scattered photons compared to the primary photons is increased. This problem is inherent to the cone-beam design, since collimating the photons cannot be as strict as in fan-beam CT scanners. Truncation artifacts are deteriorating the image quality further, see e.g. the outer body contour of patient 4 in fig. 3.

### Correction of the target point

In all evaluated cases the threshold of 2 degree rotation was not violated. The detected maximum setup deviation was 3 mm for patients immobilized with the body frame, and 6 mm for patients positioned on a vacuum pillow. Due to the action level of 2 mm translation, a target point correction was carried out in 4 cases (table 2). The additional workload of the described workflow compared to a normal treatment fraction led on average to an extra time of about 10–12 minutes (table 3).

**Table 2:** Setup deviations evaluated with CBCT

**Patient number**	**latero-lateral shift**	**ventro-dorsal shift**	**cranio-caudal shift**	**max. rotation**	**target point correction**	**image quality**
#1	3.1 mm	0.1 mm	6.0 mm	0°	yes	good
#2	-0.6 mm	0.5 mm	-0.7 mm	0.6°	no	good
#3	-0.7 mm	-1.2 mm	2.3 mm	0.7°	yes	poor
#4	1.2 mm	3.6 mm	0.1 mm	1.1°	yes	sufficient
#5	0.3 mm	0.1 mm	0.1 mm	0°	no	sufficient
#6	-2.6 mm	-1.7 mm	-1.7 mm	1.5°	yes	sufficient

**Table 3:** Mean time intervals needed for cone-beam CT setup evaluation

**Patient number**	**data aquisition **[min:sec]	**image reconstruction **[min:sec]	**image correlation **[min:sec]	**setup evaluation **[min:sec]	**total time **[min:sec]
#1	02:10	03:20	04:20	01:40	11:30
#2	02:02	03:12	04:10	02:20	11:44
#3	02:20	02:46	04:00	01:10	10:16
#4	02:00	02:40	02:30	00:20	07:30
#5	02:00	02:37	04:10	01:50	10:37
#6	02:00	03:15	03:10	01:40	10:05

**mean**	**02:05.3**	**02:58.3**	**03:43.3**	**01:30.0**	**10:16.9**

### Dealing with rotational errors

In this work, we implemented rigid matching (detecting translational and rotational errors) and correction of tranlational errors only into the clinical workflow. In cases where the rotations exceed the threshold, it might be helpful to temporarily losen the patient's fixation and reposition him with the observed deviation in mind (e.g. advising him to lift one shoulder for a rotation along the body axis). Then the workflow would start again with the acquisition of a new 3D image data set. This would add another 3–5 minutes to the workflow. Another approach for better compensation of rotational errors would be not only to shift the patient but also to modify the gantry, collimator and couch angle [12].

### Prostate cancer

There is fairly strong evidence that at least patients with localized prostate cancer with intermediate to high risk benefit from higher than conventional prescribed total dose values [13]. There is some evidence that 3D conformal radiotherapy results in reduced late rectal toxicity and acute anal toxicity compared with radiotherapy administered with non-conformal treatment volumes [14]. Ghilezan et al. have demonstrated the potential benefit of image guided radiotherapy (IGRT) for prostate cancer [15]. They have found that the ideal maximum dose increment achievable with online IGRT is, on average, 13% with respect to the dose-limiting organ of rectum. The theoretical gain of IGRT can only be achieved when organ motion/deformation can be visualized in a reasonable image quality and extra time.

In a first step of registration, we gave preference to bony landmarks of the pelvic region and calculated a target shift. We verified the correct position of the prostate and seminal vesicles after shifting the target point by visually assessing the image data sets. In the presented cases, there was a sufficient match (not more than 2 mm of deviation) between the prostate visible on the CT for treatment planning and the actual cone beam CT. In both cases, the irradiation could be started as intended. The process of soft tissue comparison is slightly hampered by the reduced image quality of the cone beam CT compared to the diagnostic CT. If an additional shift of the prostate would have been visible compared to the shift of bone structures, this would have been corrected manually. The manual match of soft tissue is a little elaborate due to the low contrast of the CT slices. Nevertheless, the entire procedure can be carried out within a reasonable time frame. The extra time needed is in the range of 8–10 minutes for image acquisition and setup evaluation and is prolonged for additional 2–3 minutes if a setup correction is necessary. We are currently working on matching algorithms that will enable the automatic correction of interfractional displacements of the prostate itself. Here, an improved image quality is necessary especially for obese patients.

### Head-and-neck tumors

In the presented case, artifacts in the cone beam images were visible close to the plane of the head-ring of the patient fixation, where significant data loss occurred due to attenuation, and in the plane of metallic implants. Nevertheless, the image quality was sufficient to work out bone structures in almost all CT slices. Since the soft tissue of the target volume is entirely framed by bone structures, the correlation of bones is sufficient for detection of setup deviations which can be carried out for the entire data set.

The target volume for head-and-neck tumors regularly includes the base of skull and extends to the upper thoracic aperture. The patients are fixated with a head mask and a vacuum pillow. The cranial part inside the head mask is very accurately repositioned during the whole treatment course. However, the location of the lower extracranial part shows more variations. The result is a complex deformation of the target volume that cannot be described by a simple translation and cannot be easily corrected by shifting the target point without changing the treatment plan [16]. In first approximation the transformation can be separated into a (small) translation of the base of skull and a rotation. Prerequisite for this procedure is that the isocenter is near the base of skull. Since available treatment tables can only be rotated within the table plane, only this rotation can be compensated. In the presented case, neither a translation nor a rotation needed to be corrected.

For high level adaptivity, the complex deformation of the target in head-and-neck irradiation can not be ignored. Changes of the patient's anatomy during the treatment course like weight loss or tumor response are common which require repeated treatment planning. Here, algorithms for automatic deformation of images and structures and automatic adaption of dose distribution by deformation of treatment fields and intensity maps are desirable. First promising approaches were presented by Mohan et al. [17] and Hansen et al.[18]

### Lung cancer

Dose escalation seems to be a useful strategy in treating non small cell lung cancer. A simple increase in the dose by giving additional fractions is limited by the tolerance doses of the surrounding tissue. The use of 3D-conformal radiotherapy significantly reduces doses to the spinal cord, heart and esophagus but does not improve lung sparing [19]. Lung has been identified as the dose limiting organ at risk in dose escalation trials [20]. Thus, dose escalation should be combined with the reduction of treatment volumes which implies a reduction of margins. Optimal would be the elimination of interfraction and intrafraction organ motion with the objective of minimizing the margins of the planning target volume. The cone beam CT allows for detection and correction of target position and for tumor tracking. In the present study we used the cone beam CT for correction of setup deviations. The high contrast of the circumscribed tumor and the surrounded lung tissue enabled manually matching of the tumor in the cone beam CT with the tumor in the CT for treatment planning.

Using the mutual information matching algorithm to match the two data sets can result in a different registration where the tumor volumes might not match. This is due to the nature of the mutual information algorithm. Therefore a manual matching method with special care given to the tumor volumes was preferred.

We are currently working on a method for respiration-triggered acquisition of cone beam CT slices by means of a belt sensor. This technique would enable gated IMRT correlated with respiration-triggered on-line fluoroscopy [21].

### Paraspinal targets

Radiotherapy of tumors near the spine is a challenge when the required total dose exceeds the tolerance of the myelon. This is the case for malignant processes like chordoma and sarcoma, but also for metastases in case of re-irradiation when the myelon tolerance was reached by the first irradiation. In the presented cases, the myelon was spared while the surrounding tumor has to be provided with high doses. Thus, a highly precise target positioning is mandatory for paraspinal targets.

On the positive side, targets near the spine are scarcely affected by intrafraction organ motion e.g. due to breathing, and a substantial distortion of the target structures does not have to be taken into account [16]. Therefore, the on-line setup registration can be limited to the matching of bone structures. The structures of interest are clearly visible in both the CT for treatment planning and the cone beam CT in treatment position. Setup deviations can be corrected by simply shifting the target point.

### General considerations

In this paper the first clinical applicaton of adaptive radiotherapy using an in-line cone beam CT attached to the linear accelerator was presented. We developed and tested a method for on-line target setup detection and correction for different tumor sites. The treated patients suffered from prostate, head-and-neck, paraspinal and thoracic tumors. The applied repositioning procedure was adapted to the special requirements for each tumor site. The additional workload of the described workflow compared to a normal treatment fraction leads in average to an extra time of about 10–12 minutes, which allows for clinical application of the process when high precision is recommended due to steep dose gradients. Partly responsible for the variation of the time values for image registration and setup deviation is the limited experience with the new hardware and software components. The total time for the entire process will most likely be reduced further by streamlining the different steps. The mutual information registration algorithm is relatively time consuming and might be replaced by a cross correlation registration algorithm in suitable cases. The image acquisition time is correlated with the speed of the gantry rotation which is actually limited to reduce any collision risk. Here, further shortening of the process seems to be possible. It seems realistic that the entire process of cone beam set up evaluation can be limited to 5 minutes.

## Conclusion

The cone beam CT attached to a LINAC allows the acquisition of a CT scan in treatment position just before treatment in sufficient image quality. The presented workflow allows target point correction in a reasonable amount of extra time, which might make sophisticated patient fixation techniques dispensable. As a result of the in-line geometry, this technology has the additional potential of being used for fluoroscopic tracking and targeting.

## Authors' contributions

CT and CTK participated in the patient treatment and drafted the manuscript. CT, UO and SN conceived of the study. TT, BH and LD participated in the design of the mounting devices and the detector. TT, SN and RB provided the used software tools, AH carried out the image registration and matching. SN, BR and PH were in charge of the approval procedure and carried out the quality assurance. JD, UO, PH participated in the study design and coordination. All authors read and approved the final manuscript.
